# Health-related quality-of-life among patients with premature ovarian insufficiency: a systematic review and meta-analysis

**DOI:** 10.1007/s11136-019-02326-2

**Published:** 2019-10-16

**Authors:** X. T. Li, P. Y. Li, Y. Liu, H. S. Yang, L. Y. He, Y. G. Fang, J. Liu, B. Y. Liu, J. E. Chaplin

**Affiliations:** 1 Institute of Basic Research in Clinical Medicine, China Academy of Chinese Medical Sciences, Beijing, China; 2 Department of Pediatrics, The Queen Silvia Children’s Hospital, Institute of Clinical Sciences, Sahlgrenska Academy, University of Gothenburg, 416 85 Gothenburg, Sweden; 3 Institute of Acupuncture and Moxibustion, China Academy of Chinese Medical Sciences, Beijing, China

**Keywords:** Surveys and questionnaires, Menstruation disturbance, Gynaecology, Women’s health

## Abstract

**Purpose:**

To systematically review studies investigating health-related quality-of-life (HrQoL) in patients with premature ovarian insufficiency (POI), to examine questionnaires used and to conduct a meta-analysis of control studies with normal ovarian function.

**Methods:**

Data sources: PubMed, Embase, Web of science, CNKI, and CQVIP, searched from inception until June 2018. The search strategy was a combination of medical (e.g. POI), subjective (e.g. well-being) and methodological (e.g. questionnaires) keywords. PRISMA guidelines were used to assess outcome data quality/validity by one reviewer, verified by a second reviewer. Risk of bias within studies was evaluated. A meta-analysis compared HrQoL in patients and non-patients. Due to measurement differences in the studies, the effect size was calculated as standard mean difference.

**Results:**

We identified 6869 HrQoL studies. Nineteen geographically diverse studies met inclusion criteria, dated from 2006, using 23 questionnaires. The meta-analysis included six studies with 645 POI participants (age 33.3 ± 5.47) and 492 normal-ovarian control subjects (age 32.87 ± 5.61). Medium effect sizes were found for lower overall HrQoL (pooled SMD = − 0.73, 95% CI − 0.94, − 0.51; *I*^*2*^ = 54%) and physical function (pooled SMD = − 0.54, 95% CI − 0.69, − 0.39; *I*^2^ = 55%). Heterogeneity was investigated. Effect sizes varied for sexual function depending on the measure (SMD = − 0.27 to − 0.74), overall HrQoL (SF-36) had the largest effect size (− 0.93) in one study. The effect sizes for psychological and social HrQoL were small.

**Conclusion:**

POI is associated with low-to-medium effect size on HrQoL compared to normal ovarian controls. The greatest effects are found in general HrQoL and most sexual function areas. Condition-specific questionnaires and RCTs are recommended for further investigation.

**Electronic supplementary material:**

The online version of this article (10.1007/s11136-019-02326-2) contains supplementary material, which is available to authorized users.

## Introduction

Thanks to medical advances, the living condition of women with premature ovarian insufficiency (POI) has gained more attention in recent years [1]. POI is a clinical syndrome defined by loss of ovarian activity before the age of 40, associated with menstrual disturbance, raised gonadotropins and low estradiol [2]. Although proper diagnostic accuracy in POI is lacking, the European Society of Human Reproduction and Embryology (ESHRE) has developed guidelines on management of women with premature ovarian insufficiency [2] in which they recommend the following diagnostic criteria for POI: (i) oligo/amenorrhea for at least 4 months, and (ii) an elevated FSH level > 25 IU/l on two occasions > 4 weeks apart. The nomenclature has changed over the years and POI has been referred to as premature ovarian failure, premature menopause, and premature ovarian dysfunction [3]. Earlier studies often used the term premature ovarian failure (POF) and more recent articles have used POI. It should also be noted that in POI serum follicle-stimulating hormone (FSH) levels are often found to exceed the diagnostic definition in studies of POI and are noted in several studies to be above 40 IU/L [2–4]. An earlier study reported the prevalence of POI in women under 30 years old estimated to be 0.1%, while the incidence of menopause in women before the age of 40 is approximately 1% [5]. In recent years, studies have investigated the prevalence of patients with POI in different countries. For example, one article reported a higher prevalence (1.9%; 95% CI 1.7–2.1) of POI in women before the age of 40 in Sweden [6] and another article reported 0.91% (95% CI 0.81–1.02%) in Estonia [7]. There has been a long-standing confusion over the various terms such as poor ovarian responders (POR), premature menopause and diminished ovarian reserve (DOR) [2, 3, 8, 9]. It is important to distinguish these conditions from POI because women with POI face more challenges than diminished fertility, and have different management needs [2, 10]. Only 5–10% of women with POI may be able to spontaneously conceive and deliver a child [11]. In addition, women with POI suffer from amenorrhea-related symptoms [12] psychological problems [13, 14], increased risk to cardiovascular health [15, 16] and to bone health [17]. POI is a condition that is influenced by genitourinary and sexual function [18] and neurological dysfunction [19] in both the short- and long-term and can lead to premature death [20]. The best option to relieve symptoms and protect POI patients against serious morbidity related to prolonged estrogen deficiency is hormone replacement therapy (HRT). However, HRT is just a mimic of normal physiological endocrinology, which has no evidence to improve the ovary function [2]. Consequently, patients with POI are at risk of poor health quality despite available treatment options. Quality of life (QoL) is a broad multidimensional concept that usually includes subjective evaluations of both positive and negative aspects of life [21]. While, health-related quality of life (HrQoL) focus on the effects of a disease on an individual’s health and its treatment [22–25] encompassing physical, psychological, and social functioning [23, 26] and presents an avenue for the evaluation of the consequences of experiencing premature ovarian insufficiency. This review aimed to investigate studies of women with POI, which have included measures of HrQoL, in order to evaluate effect sizes and in addition to identify the measurement instruments used. A meta-analysis was conducted of the studies that reached quality standards and which compared the HrQoL outcomes among patients with POI with a control group consisting of normal ovary function women.

## Materials and methods

This study followed the Preferred Reporting Items for Systematic Reviews and Meta-analyses (PRISMA) [27] reporting guideline (Online Resource ESM_1). A submission to the ethics committee of the Clinical Basic Medicine Institute, China Academy of Chinese Medical Sciences was sought. The Ethics committee judged that ethical approval was not required for this research (ref 2019/1).

### Search strategy and data selection

**Fig. 1:**
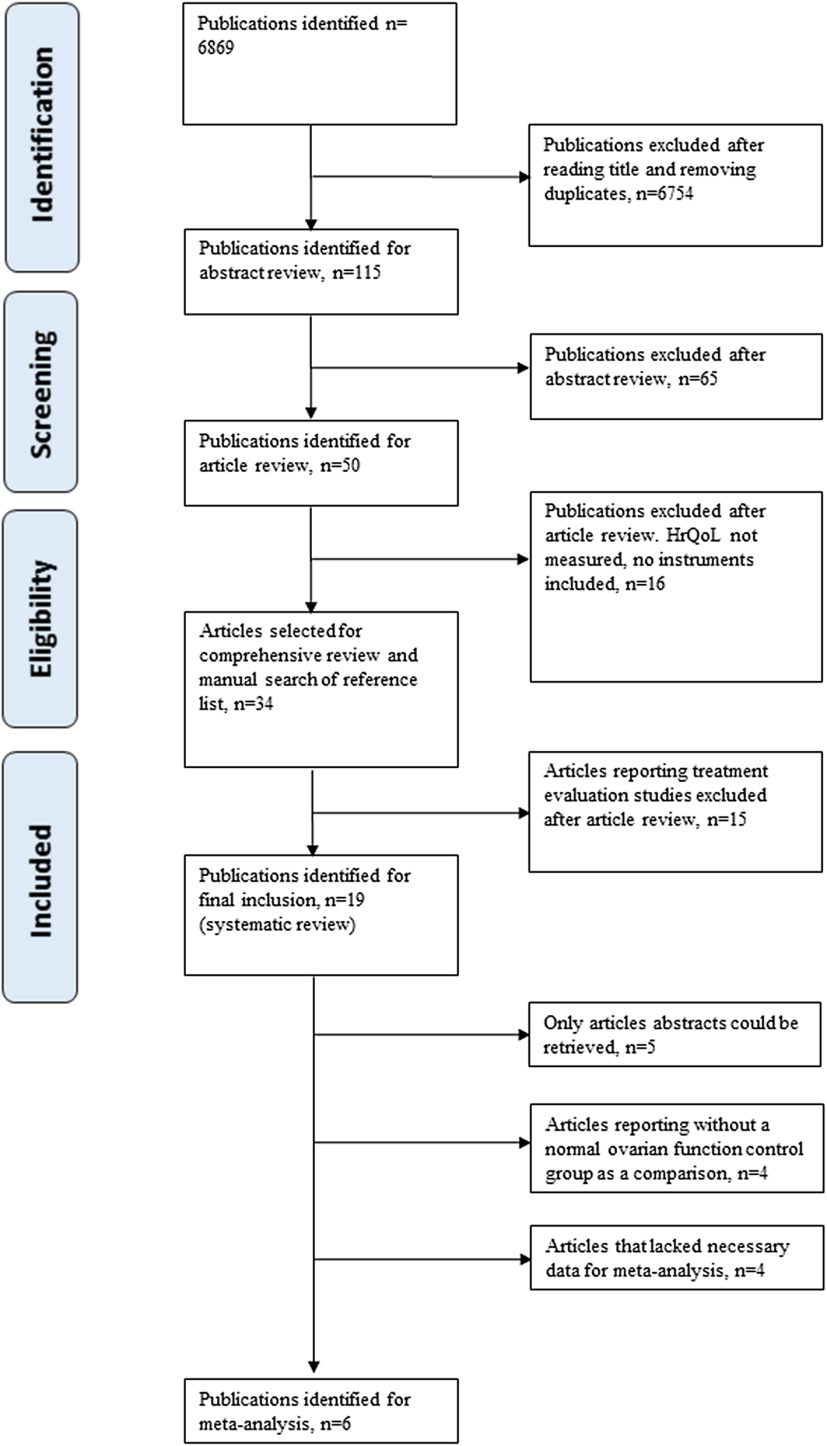
The article selection process and criteria for selection for the literature review and meta-analysis

An electronic search of the six databases was undertaken from database inception to June 2018. PubMed/MEDLINE and ‘Web of science’ provided a broad coverage of the biomedical literature, including reproductive biology and clinical medicine. EMBASE was included because it has greater coverage of European and non-English language publications and topics such as alternative medicine. China National Knowledge Infrastructure (CNKI), WanFang database and Chongqing VIP information (CQVIP) were included to ensure that no Asian publications were missed. Searches were conducted without restrictions with respect to publication year, language, type or setting of study or accessibility to full-text articles. A combination of keywords and database specific terms was used (premature ovarian insufficiency OR premature ovarian failure OR diminished ovarian reserve OR poor ovarian response OR premature menopause OR hyper-gonadotropic hypogonadism OR elevated gonadotrophins OR triad of amenorrhea OR estrogen deficiency) AND (well-being OR health outcome OR quality-of-life OR health-related quality of life) AND (questionnaire OR instrument OR patient reported outcome). Strategies differed in the different databases depending upon the information structures. The details of the different search strategies are provided in the online resource materials (online resource ESM_2). The process of article selection is outlined in Fig. 1 with a description of predefined criteria for selection. One author (XT Li) was mainly responsible for screening the titles and abstracts. Articles identified were independently read and discussed with two more authors (HS Yang, PY Li) to ensure an unbiased selection. Some studies of post-menopause have used instruments such as the MSQOL [28, 29] however this is not a measure of subjective quality-of-life and was therefore not included in this review. No additional articles were identified through the manual search. Studies describing the construction and validity of the HrQoL questionnaires used in the studies were also evaluated. If information on construction and validity was sparse, contact was attempted with the author responsible for the development of the questionnaire.

### Criteria to select articles

The inclusion criteria for empirical investigation studies of adults with POI was that HrQoL was a primary or secondary outcome. Studies with participants from hospitals and long-term care facilities or with specific conditions (e.g. Turner syndrome or anorexia) or where abstracts only were found were included in the literature in order to be able to extract data on the questionnaires used but excluded from the meta-analysis. No restrictions were placed on the geographic, soioeconoimic or ethinic backgrounds of any of the participants. There was no restriction in terms of treatment, both randomized and non-randomized trials were included. Exclusion criteria for the systematic review were duplicate publications or reviews, studies that did not include outcomes from a HrQoL questionnaire. Exclusion criteria for the meta-analysis were articles which lacked relevant data for investigation and studies without a normal ovary function control group.

## Critical appraisal: assessment of bias in the studies

The quality of eligible articles was assessed at the study level using the Newcastle–Ottawa Scale (NOS) for nonrandomized cohort studies [30]. Each article was awarded a ‘star’ or score out of four for selection bias, two for comparability and three for bias in the outcome assessment, with a maximum total score of nine points. The NOS score was used to assess differences in study quality scores > 6 high; 4–6 medium, < 4 low [31]. The scoring system and evaluation is provided in the Online Resource ESM_3. Two authors (XT Li, PY Li) independently evaluated the findings of each study to ensure an unbiased assessment.

## Meta-analysis

A meta-analysis investigated the outcome of HrQoL in patients with POI compared with a normal ovary function reference population. Review Manager (Version 5.3. Copenhagen: The Nordic Cochrane Centre, The Cochrane Collaboration, 2014) was used. The estimated value and 95% confidence interval (95% CI) of the effect size was calculated by Standard Mean Difference (SMD) [32]. The SMD is used as a summary statistic in meta-analysis when the studies all assess the same outcome but measure it in a variety of ways [33]. Cohen [34] suggested that d = 0.2 be considered a ‘small’ effect size, 0.5 represents a ‘medium’ effect size and 0.8 a ‘large’ effect size. The size of heterogeneity among studies after combination was determined via *I*^2^ statistic: 0% to 40%: might not be important; 30% to 60%: may represent moderate heterogeneity; 50% to 90%: may represent substantial heterogeneity; 75% to 100%: considerable heterogeneity [35]. If there was no heterogeneity among studies, a fixed effects model was applied for meta-analysis; if there was statistical heterogeneity, the sources of heterogeneity were further analyzed, and a random effects model was adopted for meta-analysis. According to the same questionnaires used and same specific domain evaluated, the effect sizes were divided into subgroups. This systematic review and meta-analysis were performed and reported according to the PRISMA guidelines. The PRISMA checklist is included as Online Resource_3.

## Results

Thirty-four studies matched the inclusion criteria and were included for review. Fifteen articles were related to treatment evaluation while 19 articles examined elements of HrQoL (Tables 1, 2). In five of these studies only the abstracts were available for examination [36–40]. These articles were all published between 2006 and 2018. Eighteen articles were cross-sectional studies [36–53] two of which included case–controls [43, 51]. One article reported only case–control data [54]. Nine articles described HrQoL among patients with the nomenclature of POI [36, 39, 40, 42, 47, 49, 51–53] and ten articles described HrQoL among patients with the previous nomenclature of POF [37, 38, 41, 43–46, 48, 50, 54]. Thirteen articles had control groups [39–46, 48, 49, 51, 53, 54] and nine of these had a control group of women with normal ovarian function [41–46, 51, 53, 54], six of these had sufficient information to be included in the meta-analysis [41–45, 54]. None of the studies used proxy-reports from family members as part of the evaluation. Reported studies had varying sample sizes; the largest sample size was 340 women [46]. The studies were geographically diverse including China [41, 44–46], UK [37, 38, 50], America [36, 39, 40, 42, 49, 51–53], Brazil [43, 54], Australia [48] and multi-national studies [47] (Fig. 1 and Tables 1, 2).

**Table 1:** Presentation of details of studies included in the systematic review and included in the meta-analysis

Author, year [Ref]/country	Title	Type of study	Objective of the study	Questionnaire [ref]/type of questionnaire	Sample size/observation group (age range) and population	Control group (size), mean (SD) and population	NOS
Pang et al. 2007 [41]/China	Investigations of personality characteristics and mental health status in patients with premature ovarian failure	Cross-sectional study	Analysis of personality characteristics and mental health status of patients with premature ovarian failure	TABP/TCBP [57–59]/Behaviour pattern	*N* = 80 no description of age range Hospital-based	PCOS *N* = 80, Normal *N* = 81 no description of age range Population-based	7 High
Kalantaridou et al. 2008 [42]/USA	Sexual function in young women with spontaneous 46, XX primary ovarian insufficiency	Cross-sectional study	To assess sexual function in women with spontaneous 46, XX primary ovarian insufficiency after at least 3 months of a standardized hormone replacement regimen	DISF-SR-Female Version/[60, 61]/sexual function	*N* = 143 32 ± 5.5 years Hospital-based	Women of healthy, nonpregnant, and regularly menstruating *N* = 70 28.5 ± 7.3 years Population-based	7 High
Benetti-Pinto et al. 2011 [43]/Brazil	Quality of life in women with premature ovarian failure	Cross-sectional and Case–control study	Evaluate quality-of-life in women with a diagnosis of premature ovarian failure (POF)	WHOQoLBREF-100/[62–64]/Generic QoL	*N* = 58 22–39 years 44.8%, 40–51 years 55.2% Hospital-based	Women with normal ovarian function *N* = 58 22–39 years 53.4% 40–51 years 46.6% Hospital-based	7 High
Ji 2013 [44]/China	Clinical study on the relationship between syndrome types differentiation of TCM and quality-of-life in premature ovarian failure	Cross-sectional study	To understand the quality-of-life in patients with premature ovarian failure and to explore the correlation between TCM syndrome types and quality of life	SF-36/[65–67]/Generic QoL	*N* = 114 34.5 ± 3.66 years Hospital-based	Women with normal ovarian function *N* = 90 34.6 ± 3.2 years Hospital-based	7 High
Yang et al. 2017 [45]/China	Study on quality of fertility in patients with premature ovarian failure	Cross-sectional study	Investigation of reproductive quality-of-life in patients with premature ovarian failure	FertiQoL/[68, 69]/Fertility specific	*N* = 170 31.2 ± 5.8 years Hospital-based	women with normal ovarian function *N* = 113 30.5 ± 5.3 years Hospital-based	7 High
Yela et al. 2018 [54]/Brazil	Influence of sexual function on the social relations and quality of life of women with premature ovarian insufficiency	Case–control study	To evaluate the impact of sexual function (SF) in the quality-of-life of women with premature ovarian insufficiency (POI)	1. FSFI/[70–72]/Sexual function 2. WHOQoL-BREF [62–64]/Generic QoL	*N* = 80 38.4 ± 7.3 years Hospital-based	women matched by age (± 2 years) and presenting preserved gonadal function free of chronic diseases *N* = 80 38.1 ± 7.3 years Hospital-based	7 High

**Table 2:** Studies included in the systematic review not included in the meta-analysis due to insufficient data or non-normal ovarian function control group

Author, year [ref], country	Title	Type of study	Objective of the study	Questionnaire	Sample size/observation group (age range) and population	Control group(size) and population	NOS
Pang 2006 [46], China^a^	The demonstration study of the relationship between the social/psychology factors in patients with POF	Cross-sectional study	To study the relationship between premature ovarian failure and psychosocial factors such as emotional state, personality characteristics and negative life events	1. TABP/TCBP (reported 2007) 2. STAI 3. Life Events Scale	*N* = 80 33.3 ± 5.33 years Hospital-based	PCOS *N* = 60 25.6 ± 4.7 years, Normal *N* = 200 33.53 ± 5.29 years Population-based Insufficient data reported	8 High
Davis et al. 2010 [51], USA	The psychosocial transition associated with spontaneous 46, XX primary ovarian insufficiency: illness uncertainty, stigma, goal flexibility, and purpose in life as factors in emotional health	Cross-sectional and case–control study	To examine factors associated with emotional well-being in women with spontaneous primary ovarian insufficiency	1. CES-D 2. STAI 3. PANAS 4. Purpose in Life	*N* = 99 32.4 ± 5.2 years Hospital-based	Healthy control women of similar age *N* = 60 31.0 ± 6.9 years Population-based Insufficient data reported	7 High
Orshan et al. 2009 [53], USA	Women with spontaneous 46, XX primary ovarian insufficiency (hypergonadotropic hypogonadism) have lower perceived social support than control women	Cross-sectional study	To test the hypothesis that women with spontaneous POI differ from controls regarding perceived social support and to investigate the relationship with self-esteem	1. PRQ85 2. Rosenberg’s Self Esteem Questionnaire	*N* = 154 32.2 ± 4.9 years Hospital-based	Control women: healthy, free of chronic disease, not pregnant, and regularly menstruating *N* = 63 29.9 ± 7.0 years Population-based Insufficient data reported	7 High
Gibson-Helm et al. 2014 [48], Aus	Symptoms, health behavior and understanding of menopause therapy in women with premature menopause	Cross-sectional study	To explore symptoms, understanding of menopausal therapies, medication use and health-related behavior in women with and without premature menopause	GCS	*N* = 25 36 ± 8.0 years Population-based	Premenopausal women *N* = 23, 29 ± 13 years and women with medically induced premature menopause (MIPM)*N* = 29 38 ± 4.0 years Population-based	6 Medium
Schmidt et al. 2011 [49], USA	Depression in Women with Spontaneous 46, XX Primary Ovarian Insufficiency	Cross-sectional study	To characterize the prevalence of psychiatric disorders and the onset timing of clinically significant depression relative to POI and the onset of menstrual irregularity in women with POI	[DSM-IV] (SCID)	*N* = 174 31.6 ± 5.3 years Hospital-based	Turner syndrome *N* = 100 no description of age range Hospital-based	3 Low

^a^English translations of the Chinese abstracts are included as Online Resources ESM_4

## Domains of HrQoL examined

The definition of HrQoL used in the studies is derived from the domains of the questionnaires used to measure HrQoL. Among the 19 articles examining HrQoL, seven studies included a measure of overall HrQoL as measured by either a generic questionnaire (SF-36, WHOQoL-BREF) [37, 43, 44, 50, 54] or measured in relation to fertility or sexual function [42, 45, 50, 54]. Nine studies focused on psychiatric aspects including depression and meaning in life [36, 38–40, 49–53]. Four articles used the POI related symptom questionnaires [38, 47, 48] Only one of these [50] used a condition specific instrument designed for POI (Young Menopause Assessment (YMA) [50]). One study evaluated the aspect of social function: perceived social support [53]. The reduced HrQoL among patients with POI was mentioned in all 19 articles. A summary of the studies is found in Tables 1, 2.

## Overall HrQoL

Three articles described factors correlated with lower HrQoL in POI populations: one article reported that orgasm and sexual satisfaction were correlated with all QOL domains [54]; a second article analysed character traits of POI patients [45], which showed that older patients, with primary infertility and who had had children had lower HrQoL scores than patients who were of younger age, secondary infertility or had previously given birth. In one article [44] different Traditional Chinese Medicine (TCM) syndromes were considered as summaries of symptoms of the pathogenesis of disease development [55]. These syndromes included insufficiencies of liver and kidney or asthenia of both the spleen and kidney. It was noted that patients with deficiency of liver and kidney had the lowest overall QOL scores (Table 3).

**Table 3:** Studies included in the systematic review not included in the meta-analysis due to insufficient data and no control group

Author/year, country	Title	Type of study	Objective of the study	Questionnaire	Sample size/Observation group (age range) and population	Control group(size) and population
Allshouse et al. 2014 [47], USA + International	Evidence for prolonged and unique amenorrhea-related symptoms in women with POF/POI	Cross-sectional study	Aims to describe POF/POI symptoms experienced by women from members of a POF/POI-specific support group	1. Menopause-specific QoL + 10 symptoms 2. CAMS-R	*N* = 160 39.3 ± 7.3 years Population-based	No control group
Singer et al. 2011 [50], UK	The silent grief: psychosocial aspects of premature ovarian failure	Cross-sectional study	To investigate experiences of diagnosis, perception of cause, treatment, concerns, a self-esteem, sexual functioning and HrQoL	1. Rosenberg’s Self Esteem 2. SF 36; 3. YMA; 4. SPEQ	*N* = 136 38.7 ± 7.03 years Hospital-based	No control group
Ventura et al. 2007 [52], USA	Functional well-being is positively correlated with spiritual well-being in women who have spontaneous premature ovarian failure	Cross-sectional study	To examine the relationship between spiritual well-being and functional well-being in women who have spontaneous POF	1. FANLTC 2. FACIT-Sp-12	*N* = 137 32 years Hospital-based	No control group
Sterling et al. 2009 [36], USA	A study of the relational aspects of spiritual well-being and functional well-being in women with spontaneous 46, XX POI	Cross-sectional study	To analyze the relational aspects of spirituality and functional well-being in women with spontaneous 46, XX sPOI	1. FACIT-Sp-Ex 2. FANLTC	*N* = 140 No description of age range Source unreported	No control group Abstract only
Islam et al. 2011 [37], UK	The impact of premature ovarian failure on quality of life: results from the UK 1958 Birth Cohort	Cross-sectional study	To assess the prevalence and quality-of-life impact of premature ovarian failure in a large population based sample	SF-36	*N* = 370 No description of age range Population-based	No control group Abstract only
Nicopoullos et al. 2009 [38], UK	Effect of age and aetiology of premature ovarian failure on symptoms at presentation data from the west London POF database	Cross-sectional study	To assess the effect of age at diagnosis and aetiology on presentation	Symptom questionnaire(no details)	*N* = 239 No description of age range Hospital-based	No control group Abstract only
Covington et al. 2009 [39], USA	Perceived mastery and emotional well-being in women with 46, XX primary ovarian insufficiency	Cross-sectional study	To compare mastery in women with 46, XX sPOI to controls and assess associated affective symptoms	1. Pearlin Mastery Scale 2. CES-D; 3. STAI; 4. PANAS	*N* = 100 No description of age range Source unreported	Control women *N* = 60 no description of age range Source unreported Abstract only
Vanderhoof et al. 2009 [40], USA	Spirituality and emotional well-being in women with spontaneous 46, XX primary ovarian insufficiency (SPOI)	Cross-sectional study	To compare spirituality and religiousness of women with sPOI to controls, and assess the association with affective symptoms	1 Spirituality and Religion 2. CES-D; 3. STAI; 4. PANAS	*N* = 100 No description of age range Source unreported	Control women *N* = 60 no description of age range Source unreported Abstract only

## Physical function and symptoms

Physical health of the women with POI was consistently reported to be significantly lower than controls. A number of physical function symptoms were explored including experience of physical pain [43] sexual function [42, 54] arousal, lubrication, orgasm and satisfaction, and sexual behaviour/experiences [42, 50, 54]. In addition, menopause symptoms such as vasomotor symptoms, mood swings and mental fog, hair loss, dry eyes, cold intolerance, joint clicking, tingling in limbs and low blood pressure were found at a high rate in patients with POI [47].

## Psychological function and psychosocial aspects

Women with spontaneous POI were reported to score adversely on all measures of psychological functioning [43, 51] with higher negative feelings such as “blue mood” [56], despair, anxiety, and depression or had a negative impact on their self-image and confidence [50]. This population also had a high rate of mental health medication use and counselling [51] and a risk for depression [49]. Some articles analysed the factors related to these negative feelings. Adverse affective symptoms were associated with a lower perceived level of control [39]. One article reported illness uncertainty and lack of purpose in life as a significant independent factor associated with anxiety [51]. Scores on the Spiritual Well-Being scale were also associate with POI and were found to reduce with increased age [52].

## Social function

Marital relationship and social support were reported to be significantly lower in POI patients [45]. Social relationships were found to have a negative influence of sexual function such as arousal, orgasm, satisfaction and pain [53, 54]. However, other articles reported no significant differences found with respect to the social relationships or support [43, 46].

## Questionnaires

In total, twenty-three different questionnaires had been used in the nineteen articles identified for review (Table 4). The most frequently used questionnaires were the two generic HrQoL: World Health Organization Quality of Life (WHOQoL-BREF) [62–64], and the 36-Item Short Form Survey from the RAND Medical Outcomes Study (SF-36) [65–67] which were used in five studies. Between 1 and 4 questionnaires were used in each study, 50% of the studies only used one questionnaire. Those studies that used four concentrated on the psychological aspects of the condition and were mainly from the same research group at NIH in the US and reported in Abstract form. Other studies combined generic questionnaires with condition specific issues e.g. sexual or menopause specific questionnaires. Only one study [50] used a POI specific questionnaire (Young Menopause Assessment (YMA) [Unpublished]. This was used in combination with a sexual function questionnaire (Sexual Personal Experiences Questionnaire (SPEQ) [73]) a psychological questionnaire (Rosenberg’s Self Esteem Questionnaire [74–77]) and a generic questionnaire (SF-36 Short Form Survey from the RAND Medical Outcomes Study (SF-36) [65–67]). All the HrQoL instruments used are described in Table 4, a more detailed summary of the six questionnaires used in the studies included in the meta-analysis can be found as Online Resource ESM_5.

**Table 4:** Questionnaires used in the studies included in the systematic review

Focus of scale	Instrument	Instrument description	Study	Study origin
Generic HrQoL	World Health Organization Quality of Life (WHOQoL-BREF) [62–64]^a^	Last 4 weeks/5 point Likert. 4 domains: Social, Emotional, Physical, Environmental (28 items)	Benetti-Pinto 2011 [43] Yela 2018 [54]	São Paulo, Brazil São Paulo, Brazil
	SF-36 RAND Medical Outcomes Study [65–67]^a^	Last 4 weeks/5 point Likert. 8 domains: Physical, Role limitations, Bodily pain, Social, General mental health, Role limitations/emotional, Vitality, Gen health. (36 items)	Singer 2011 [50] Islam 2011 [37] Ji 2013 [44]^b^	London, UK London, UK GuangZhou, China
Functional well-being	Functional Assessment of Non-Life-Threatening Conditions (FANLTC) [78]	Last 7 days/5 point Likert 4 domains: Physical, Social/Family, Emotional, Functional (25 items)	Ventura 2007 [52] Sterling 2009 [36]	NICH, USA NIH, USA
Psychological aspects of HrQoL	The Cognitive and Affective Mindfulness Scale V Revised (CAMS-R) [79]	No time scale/4 point Likert. 1 domain: Mindfulness (10 items)	Allshouse 2014 [47]	Colorado, USA
	The Pearlin Mastery Scale (PM) [80, 81]	No time scale/7 point Likert. 1 domain: Mastery (7 items)	Covington 2009 [39]	Arizona, USA
	Epidemiologic Studies Depression Scale (CES-D) [82–84]	Last 7 days/4 point Likert 1 domain: Depression (20 items)	Covington 2009 [39] Vanderhoof 2009 [40] Davis 2010 [51]	NIH, USA NIH, USA NIH, USA
	State-Trait Anxiety Inventory (STAI) [85–88]	At the moment/4 point Likert. 2 domains: State and Trait Anxiety (40 items)	Pang 2006 [46] Covington2009 [39] Vanderhoof2009 [40] Davis 2010 [51]	GuangZhou, China NIH, USA NIH, USA NIH, USA
	Positive and Negative Affect Schedule (PANAS) [ [89–91]	Time scale appropriate to the study/5 point Likert. 1 domain: Positive/negative affect (40 items)	Davis 2010 [51] Covington2009 [39] Vanderhoof 2009 [40]	NIH, USA NIH, USA NIH, USA
	Type A behavior pattern TABP/TCBP [57–59]^a^	Current time/dichotomous. 3 domains: Time urgency, Hostility, Competitive drive (60 items)	Pang 2007 [51] Pang 2006 [46]	GuangZhou, China GuangZhou, China
	Rosenberg’s Self Esteem Questionnaire [74–77]	Current time/4 point Likert. 1 domain: Self worth (10 itmes)	Singer 2011 [50] Orshan 2009 [53]	London, UK NICH, USA
	Purpose in Life subscale from the Positive Mental Well-Being Inventory [92, 93]	Current time/7 point Unmarked Semantic Differential Scale. 1 domain: Meaning and purpose (20 items)	Davis 2010 [51]	NIH, USA
	Functional Assessment of Chronic Illness Therapy—Spiritual Well-Being Scale (FACIT-Sp-12) [94]	Last 7 days/5 point Likert. 3 domains: Spiritual well-being (peace, meaning, faith) (12 items)	Ventura 2007 [52]	NICH, USA
	Functional Assessment of Chronic Illness Therapy—Spiritual Well-Being Scale Expanded (FACIT-Sp-Ex) [94]	Last 7 days/5 point Likert. 3 domains: Spiritual well-being (peace, meaning, faith) (23 items)	Sterling 2009 [36]	NIH, USA
	Brief Multidimensional Measure of Religiousness/Spirituality [95, 96]	Current time/6-point scale. 9 domains: Daily spiritual experiences, Meaning, Values/Beliefs, Forgiveness, Religious practice, Spiritual coping, Religious support, Religious History, Commitment (40 items)	Vanderhoof 2009 [40]	NIH, USA
Life events	Life events scale(LES) [97]	No time limit/. 1 domain: Life events (48 items)	Pang 2006 [46]	GuangZhou, China
Sexual function	Female Sexual Function Index (FSFI) [70–72]^a^	Last 4 weeks/5 point Likert. 6 domains: Desire, Arousal, Lubrication, Orgasm, Satisfaction, Pain (19 items)	Yela 2018 [54]	São Paulo, Brazil
	Derogatis Interview for Sexual Function (DISF-SR—Female Version) [60, 61]^a^	Current time/9 and 5 point scales. 4 domains: Sexual cognition and fantasy; Sexual arousal; Sexual behaviour and experience; orgasm; Sexual drive and relationship (25 items)	Kalantaridou 2008 [42]	NIH, USA
	Short Personal Experiences Questionnaire (SPEQ) [73]	Current time/8 domains: Desire, Arousal, Orgasm, Enjoyment, Satisfied by frequency, Frequency of intercourse, Frequency of fantasies, Dyspareunia (9 items)	Singer 2011 [50]^b^	London, UK
Disease or symptom-specific	Fertility Quality of Life Questionnaire(FertiQoL) [68, 69]^a^	Current time/5 point Likert. 4 domains: Emotional, Mind–body, Relational; Social. (36 items)	Yang 2017 [45]	Henan, China
	Menopause-specific Quality of Life questionnaire [28, 29, 98]	4 Weeks/7 point Likert 5 domains: Physical; Vasomotor; Psychosocial; Sexual; working life (30 items)	Allshouse 2014 [47]	Colorado, USA
	Greene Climacteric Scale (GCS) [99–101]	Symptoms checklist (21)	Gibson-Helm 2014 [48]	Monash, Australia
POI specific	Young Menopause Assessment (YMA) [50]	3 Domains: Description of POF; Treatment; information and support (3 items 6) (Designed for this study referred to as developed in a pilot study—unpublished)	Singer 2011 [50]^c^	London, UK
Perceived social support	Personal Resource Questionnaire 1985, part 2 (PRQ85) [102]	Current time/7-point scale. 5 domains: Valued individual; part of a group; intimacy; nurturance; info emotional and material help + description and satisfaction with resources (25 items)	Orshan 2009 [53]	NICH, USA

^a^Six questionnaires included in the meta-analysis are further summarized in Table S5

^b^Ji gives a measure of overall HrQoL derived from the SF-36 but does not explain how this is calculated

^c^Singer refers to the measure as the Sexual Personal Experiences Questionnaire but gives a reference to the Dennerstein Short Personal Experiences Questionnaire

## Synthesis of results and risk of bias (results of meta-analysis)

**Fig. 2:**
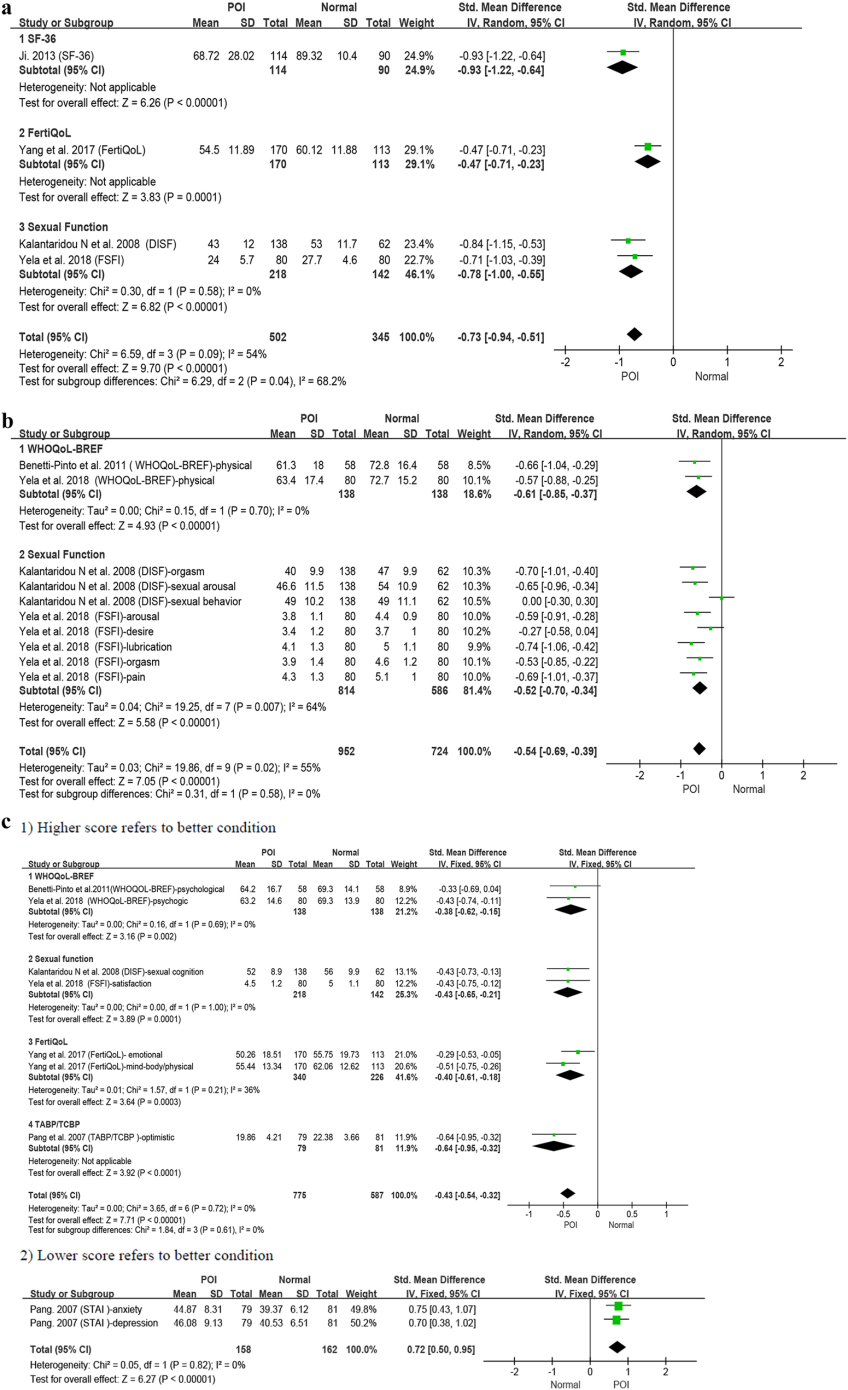

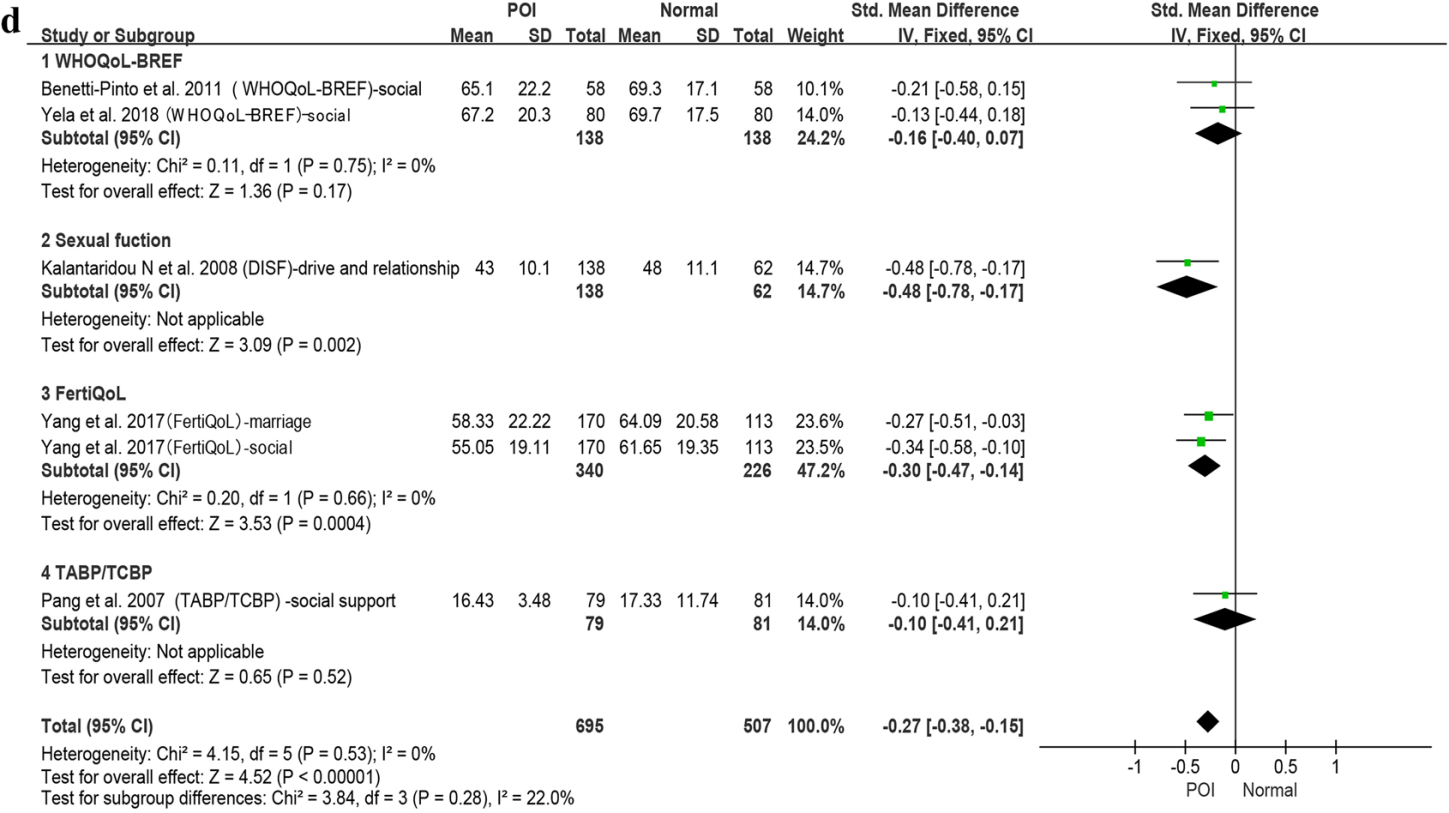
**a** Patients with POI compared with normal ovarian reference populations: overall health related quality-of-life (HrQoL). **b** Patients with POI compared with normal ovarian reference populations: physical functioning. **c** Patients with POI compared with normal ovarian reference populations: mental health. **d** Patients with POI compared with normal ovarian reference populations: social functioning

Six studies were included in the meta-analysis [41–45, 54] (Fig. 2) with 645 POI participants and 492 normal-ovarian controls. Where data on average age was available the POI group had a pooled mean age of 33.3 ± 5.47; and the control group a pooled mean age of 32.87 ± 5.61.

At the overall HrQoL level (Fig. 2a) four studies [42, 44, 45, 54] had lower level of HrQoL recorded in the POI group (pooled SMD = − 0.73, 95% CI − 0.94, − 0.51; *I*^*2*^ = 54%) as compared to a normal ovarian control group. The pooled heterogeneity can be considered moderate. To address the heterogeneity, a subgroup analysis (2 studies included) was performed to separately examine the measures of sexual functioning (Fig. 2a3) (SMD = − 0.78, 95% CI − 1.00, − 0.55; *I*^*2*^ = 0%) the effect size was medium to large and there was no indication of heterogeneity. The largest effect size (large) was found for ‘overall HrQoL’ as measured by the SF-36 (− 0.93, 95% CI − 1.22, − 0.64).

In regard to the physical functioning aspects of HrQoL (Fig. 2b), this was measured by four studies using nine different indicators. The results again showed moderate pooled effect size and moderate heterogeneity (pooled SMD = − 0.54, 95% CI − 0.69, − 0.39; *I*^2^ = 55%) as compared to a normal ovarian control group. The sexual function (2 studies included) measures explained the heterogeneity where these alone demonstrated substantial heterogeneity (*I*^*2*^ = 64%) but with a medium effect size (SMD = 0–0.52, 95% CI − 0.70, − 0.34; *I*^2^ = 64%). The largest effect size (moderate) was found for ‘Lubrication’ as measured by the FSFI (− 0.74, 95% CI − 1.06, − 0.42).

In the mental health area (Fig. 2c1, 2), the studies agreed that there was a lower level of mental health in the POI group than was found in the controls however the pooled effect size was small [1. SMD = − 0.43, 95% CI − 0.54, − 0.32; *I*^2^ = 0% (higher score = better Fig. 2c1); 2. SMD = 0.72, 95% CI 0.50, 0.95; *I*^2^ = 0% (lower score = better Fig. 2c2)]. The largest effect size (moderate) was found for ‘Optimism’ as measured by the TABP/TABC (− 0.64, 95% CI − 0.95, − 0.32).

The social functioning domain (Fig. 2d) was addressed by five of the six studies, the pooled effect size was small with no heterogeneity (pooled SMD = − 0.27, 95% CI − 0.38, − 0.15; *I*^2^ = 0%). The largest effect size (moderate) was found for ‘Drive and relationship’ in the DISF (− 0.48, 95% CI − 0.78, − 0.17).

Ji [44] has calculated a total QoL score for the SF-36. There is not information on how this was calculated. For discussion on this issue see by Lins and Martins Carvalho (2016) 10.1177/2050312116671725.

## Discussion

Nineteen studies reported the empirical measurement of HrQoL among patients with POI. Reports of the impact of POI on different aspects of HrQoL differed between studies. However, impaired physical, psychological and general health was reported across all areas of HrQoL. There were no articles prior to 2006 and studies used a variety of HrQoL instruments both generic and condition specific although only one measure was specially designed for POI [50]. Although subjective experiences of patients with POI have received more attention from the medical profession in the past decade, relevant and valid evaluation instruments have not been developed, and long-term follow-up studies of HrQoL have not been carried out.

The six controlled studies included in the meta-analysis demonstrated that overall HrQoL in patients with POI/POF is lower than individuals with normal ovarian functioning with low to medium pooled effect sizes [41–45, 54]. The moderate heterogeneity in the general measure of HrQoL appears to be due to the different concept being measured under the term HrQoL. It may also come from the different socioeconomic groups being included in the various studies. Information on socioeconomic status was sparsely reported and it was not possible for us to make an assessment of the influence of this moderator.

The finding that studies concerning HrQoL in relation to POI were not found prior to 2006 may be related to fact that the definition of POI had not been standardized. Recent guidelines from the European Society of Human Reproduction and Embryology, published in 2015 [2], coincide with the beginning of investigations into HrQoL in POI. However, some variation in diagnostic criteria is evident. Some studies used broader age intervals, and the levels of Follicle-Stimulating Hormone (FSH), which is a very important indicator of POI diagnosis [2], were vague. This may lead to heterogeneity of the results.

The factors measured in the six studies in the meta-analysis varied and included: fertility, sexual function, anxiety, depression, menopausal symptoms. Although all the measurements were cross-sectional, the concepts measures could all be considered to have long-term effects and would vary according to, for example, diagnostic age, marriage condition or education. In one study [45], an association was investigated between personal character traits and the impact of POI this highlighted the patient’s response to the stress of a POI diagnosis and of living with the condition.

Geographical diversity is apparent from our review. It is noted that studies were found in five countries and included one multi-national study [47]. Studies taking a cross-cultural perspective were not conducted. This highlights the possibility of cultural bias in the results [103]. The sparsity of these studies may be due to the lack of a single agreed and validated condition specific instrument translated into multiple language. In addition, despite substantial clinical studies on the use of traditional medicine with this condition, there is a lack of controlled studies that can be used as evidence of treatment effects.

The large number of instruments used (23) in 19 studies with a very low repetition rate, indicates that there is no common view concerning instruments. In some studies, the generic instruments were used to address a comprehensive array of domains of QoL, however, this focus may have limited the sensitivity to detect subtle aspects of POI. It is interesting to speculate on what we did not find, which was the patient perspective. The instrument designed for POI by Singer [50] for their study was based on ‘clinical experience’ and covered the areas of ‘About your POF/young menopause’, ‘Treatment’, and ‘Information and Support’. For many patients, there are concerns about the implications of the treatment and of possible long-term side effects which might be more meaningful to the patient [104, 105] and yet these aspects were not investigated. Some studies choose questionnaires that are specific for similar conditions such as menopause or infertility, however, even though the symptoms may be similar, the patients’ experiences and requirements may not be the same [47, 48, 54]. It also must be considered that these questionnaires may not be sensitive to all patients with POI. Although the majority of the questionnaires used to measure HrQoL in these studies had good psychometric properties, none of them had evidence to confirm the sensitivity and specificity of the instruments in relation to POI. There were ten studies [36, 39, 40, 46, 47, 50–54] that used a combination of questionnaires to capture more comprehensive information. However, mood, symptom, and fertility questions specific for women with POI were lacking [47, 50].

## Strengths and limitations

Some limitations of the study need to be taken into consideration. It is possible that some studies have been missed due to the use of different terms for POI or in languages that were not included in the databases we examined. There were some studies that were only published as Abstracts and although we tried to contact these researchers we were unable to obtain more information. Our study has the strength of including both European and Asian databases. Those databases that were searched are those that have the highest likelihood of finding studies of HrQoL and POI.

## Conclusion and future recommendations

This literature review and meta-analysis gives new information on HrQoL in patients with POI. In this review, the magnitude of the subjective effects is found to vary with effect sizes between low and medium. The largest effect sizes were found in the area of sexual function and general HrQoL. Cross-cultural approaches and international collaboration were found in only one study. Additional studies are recommended to make a stratified comparison of patients, larger sample sizes to identify real changes in outcomes and long-term follow-ups need to be done in order to have sufficient information for evidence based clinical practice decisions. Future research should focus on developing condition specific and sensitive assessments of the effect of POI based on the patient perspective. This can be achieved through focus groups with the aim of achieving a broader understanding of the outcome domains that are relevant to this population.

## Electronic supplementary material

Below is the link to the electronic supplementary material.

Supplementary material 1 (DOCX 20 kb)

Supplementary material 2 (DOCX 17 kb)

Supplementary material 3 (DOCX 20 kb)

Supplementary material 4 (DOCX 23 kb)

Supplementary material 5 (DOCX 25 kb)

## Abbreviations

CAMS-R: The Cognitive and Affective Mindfulness Scale V Revised
CES-D: Epidemiologic Studies Depression Scale
DHEA: Dehydroepiandrosterone
DISF-SR: Derogatis Interview for Sexual Function—Female Version
DOR: Diminished ovarian reserve
DSM-IV (SCID): Diagnostic and Statistical Manual of Mental Disorders (fourth edition)
FACIT-Sp-12: Functional Assessment of Chronic Illness Therapy—Spiritual Well-Being Scale
FACIT-Sp-Ex: Functional Assessment of Chronic Illness Therapy—Spiritual Well-Being Scale Expanded
FANLTC: Functional Assessment of Non-Life-Threatening Conditions
FertiQoL: International Fertility Quality of Life Questionnaire
FSFI: Female Sexual Function Index
FSH: Follicle-Stimulating Hormone
GCS: Greene Climacteric Scale
HrQoL: Health-related quality of life
HRT: Hormone replacement therapy
IHD: Ischaemic heart disease
LEU: Life events scale
NOS: Newcastle–Ottawa Scale
PANAS: Positive and Negative Affect Schedule
PCOS: Polycystic ovarian syndrome
PM: The Pearlin Mastery Scale
POF: Premature ovarian failure
POI: Premature ovarian insufficiency
POR: Poor ovarian responders
PRQ85: Personal Resource Questionnaire 1985
QOL: Quality of life
SF-36: The 36-Item Short Form Survey from the RAND Medical Outcomes Study
SMD: Standard Mean Difference
SPEQ: Sexual Personal Experiences Questionnaire
STAI: State-Trait Anxiety Inventory
TABP/TCBP: Type A/C behavior pattern
TCM: Traditional Chinese Medicine
WHOQoL-BREF: World Health Organization Quality of Life
YMA: Young Menopause Assessment
