# Supplementary material for: Health-related quality-of-life among patients with premature ovarian insufficiency: a systematic review and meta-analysis
Source: Qual Life Res. 2019 Oct 16;29(1):19–36. doi: 10.1007/s11136-019-02326-2 (PMC6962283; doi:10.1007/s11136-019-02326-2)
Supplement: Supplementary file 2 — Supplementary material 2 (DOCX 17 kb) [file 11136_2019_2326_MOESM2_ESM.docx]

# ESM_2 Supplementary material: Search strategy of in different databases

| Database | PubMed |
| --- | --- |
| URL | https://www.ncbi.nlm.nih.gov/pubmed/ |
| Searcher | Huisheng Yang and Xiaotong Li |
| Search strategy | #1 ("Menopause, Premature"[Mesh]) OR "Primary Ovarian Insufficiency"[Mesh]  #2 (((((((premature ovarian insufficiency[Title/Abstract]) OR premature ovarian failure[Title/Abstract]) OR diminished ovarian reserve[Title/Abstract]) OR poor ovarian response[Title/Abstract]) OR hyper-gonadotropic hypogonadism[Title/Abstract]) OR elevated gonadotrophins[Title/Abstract]) OR triad of amenorrhea[Title/Abstract]) OR estrogen deficiency[Title/Abstract]  #3 #1 OR #2  #4 ("Women's Health"[Mesh]) OR "Quality of Life"[Mesh]  #5 (((((well-being[Title/Abstract]) OR Life Quality[Title/Abstract]) OR Health-Related Quality Of Life[Title/Abstract]) OR Health Related Quality Of Life[Title/Abstract]) OR HrQoL[Title/Abstract]) OR health outcome[Title/Abstract]  #6 #4 OR #5  #7 ("Surveys and Questionnaires"[Mesh]) OR "Patient Health Questionnaire"[Mesh]  #8 ((questionnaire[Title/Abstract]) OR instrument[Title/Abstract]) OR patient reported outcome[Title/Abstract]  #9 #7 OR #8  #10 #3 AND #6 AND#9 |

| Database | Embase |
| --- | --- |
| URL | https://www.embase.com/ |
| Searchers | Huisheng Yang and Xiaotong Li |
| Search strategy | #1 ' Menopause, Premature '/exp OR ' Primary Ovarian Insufficiency '/exp  #2 'premature ovarian insufficiency ':ab,ti OR ' premature ovarian failure ':ab,ti OR ' diminished ovarian reserve ':ab,ti OR ' poor ovarian response ':ab,ti OR ' hyper-gonadotropic hypogonadism ':ab,ti OR 'reproductive techniques':ab,ti OR 'art':ab,ti OR 'blastocyst transfer':ab,ti OR ' elevated gonadotrophins ':ab,ti OR ' triad of amenorrhea ':ab,ti OR ' estrogen deficiency ':ab,ti  #3 #1 OR #2  #4 'Women's Health '/exp OR ' Quality of Life '/exp  #5 Well-being:ab,ti OR ' Life Quality '/exp OR ' Health-Related Quality Of Life ' OR ' Health Related Quality Of Life '/exp OR ' HrQoL ' OR health outcome:ab,ti  #6 #4 OR #5  #7 ' Surveys and Questionnaires '/exp OR ' Patient Health Questionnaire '/exp  #8 ' questionnaire ':ab,ti OR ' premature ovarian failure ':ab,ti OR ' diminished ovarian reserve ':ab,ti OR ' poor ovarian response ':ab,ti OR ' instrument ':ab,ti OR 'reproductive techniques':ab,ti OR 'art':ab,ti OR 'blastocyst transfer':ab,ti OR ' patient reported outcome ':ab,ti  #9 #7 OR #8  #10 #3 AND #6 AND#9 |

| Database | Web of science |
| --- | --- |
| URL | http://apps.webofknowledge.com |
| Searchers | Huisheng Yang and Xiaotong Li |
| Search strategy | #1 Topic: (Menopause, Premature) OR Topic:(Primary Ovarian Insufficiency) OR Topic:(premature ovarian insufficiency) OR Topic: (premature ovarian failure) OR Topic:(diminished ovarian reserve) OR Topic: (poor ovarian response) OR Topic:( hyper-gonadotropic hypogonadism) OR Topic: (elevated gonadotrophins) OR Topic: (triad of amenorrhea) OR Topic:(estrogen deficiency)  #2 Topic: (Women's Health) OR Topic:(Quality of Life) OR Topic:(well-being) OR Topic: (Life Quality) OR Topic:(Health-Related Quality Of Life) OR Topic:( Health Related Quality Of Life) OR Topic:(HrQoL) OR Topic:(health outcome)  #3 Topic: (Surveys and Questionnaires) OR Topic:(Patient Health Questionnaire) OR Topic:(questionnaire) OR Topic: (instrument) OR Topic: (patient reported outcome)  #4 #1 AND #2 AND #3 |

| Database | China National Knowledge Infrastructure (CNKI) |
| --- | --- |
| URL | http://www.cnki.net/ |
| Searchers | Huisheng Yang and Xiaotong Li |
| Search strategy | SU=(早发性卵巢功能不全+卵巢早衰+卵巢低反应+卵巢储备功能减退+卵巢功能+低雌激素+高促性腺激素+POR+DOR+POI+POF)*(生存质量量表+女性健康+健康相关结局+HrQoL+量表) |

| Database | WANFANG DATA |
| --- | --- |
| URL | http://www.wanfangdata.com.cn/index.html |
| Searchers | Huisheng Yang and Xiaotong Li |
| Search strategy | (主题:( 早发性卵巢功能不全)+主题:( 卵巢早衰) +主题:( 卵巢低反应) +主题:( 卵巢储备功能减退) +主题:( 卵巢功能) +主题:( 低雌激素) +主题:( 高促性腺激素) +主题:(DOR) +主题:(POR) +主题:(POI) +主题:(POF)) *(主题:( 生存质量量表) +主题:( 女性健康) +主题:( 健康相关结局) +主题:(HrQoL)+ 主题:(量表)） |

| Database | Chongqing VIP information (CQVIP) |
| --- | --- |
| URL | http://lib.cqvip.com/ |
| Searchers | Huisheng Yang and Xiaotong Li |
| Search strategy | (M=早发性卵巢功能不全+ M=卵巢早衰+ M=卵巢低反应+ M=卵巢储备功能减退+ M=卵巢功能+ M=低雌激素+ M=高促性腺激素+ M=DOR + M=POR + M=POI+ M=POF) * (M=生存质量量表+ M=女性健康+ M=健康相关结局+ M= HrQoL + M=量表) |
