# Supplementary material for: Health-related quality-of-life among patients with premature ovarian insufficiency: a systematic review and meta-analysis
Source: Qual Life Res. 2019 Oct 16;29(1):19–36. doi: 10.1007/s11136-019-02326-2 (PMC6962283; doi:10.1007/s11136-019-02326-2)
Supplement: Supplementary file 3 — Supplementary material 3 (DOCX 20 kb) [file 11136_2019_2326_MOESM3_ESM.docx]

# ESM_3 Newcastle-Ottawa Scale (NOS) evaluation

| **Title** | **Is the case definition adequate?** | **Representativeness of the cases** | **Selection of Controls** | **Definition of Controls** | **Comparability of cases and controls on the basis of the design or analysis** | **Ascertainment of exposure** | **Same method of ascertainment for cases and controls** | **Non-Response rate** | **Total** |
| --- | --- | --- | --- | --- | --- | --- | --- | --- | --- |
| Pang et al 2007 Investigations of personality characteristics and mental health status in patients with premature ovarian failure [1]. | ☆ | ☆ | ☆ | ☆ | ☆ | interview not blinded to case/control status | ☆ | ☆ | 7 |
| Kalantaridou et al 2008 Sexual function in young women with spontaneous 46, XX primary ovarian insufficiency [2] | Two FSH levels in the menopausal range, which is unclear | ☆ | ☆ | ☆ | ☆  ☆ | interview not blinded to case/control status | ☆ | ☆ | 7 |
| Benetti-Pinto et al 2011 Quality of life in women with premature ovarian failure [3] | ☆ | ☆ | hospital controls | ☆ | ☆  ☆ | interview not blinded to case/control status | ☆ | ☆ | 7 |
| Ji 2013 Clinical study on the relationship between syndrome types differentiation of TCM and quality-of-life in premature ovarian failure [4] | ☆ | ☆ | hospital controls | ☆ | ☆  ☆ | interview not blinded to case/control status | ☆ | ☆ | 7 |
| Yang et al 2017 Study on quality of fertility in patients with premature ovarian failure [5] | ☆ | ☆ | hospital controls | ☆ | ☆  ☆ | interview not blinded to case/control status | ☆ | ☆ | 6 |
| Yela, et al 2018 Influence of Sexual Function on the Social Relations and Quality of Life of Women with Premature Ovarian Insufficiency [6] | ☆ | ☆ | hospital controls | ☆ | ☆  ☆ | interview not blinded to case/control status | ☆ | ☆ | 7 |
| Pang 2006 The demonstration study of the relationship between the social/psychology factors in patients with POF [7]. | ☆ | ☆ | ☆ | ☆ | ☆  ☆ | interview not blinded to case/control status | ☆ | ☆ | 8 |
| Davis et al 2010 The psychosocial transition associated with spontaneous 46, XX primary ovarian insufficiency: illness uncertainty, stigma, goal flexibility, and purpose in life as factors in emotional health [8]. | The diagnosis criteria were unclear | ☆ | ☆ | ☆ | ☆  ☆ | interview not blinded to case/control status | ☆ | ☆ | 7 |
| Orshan et al 2009 Women with spontaneous 46, XX primary ovarian insufficiency (hypergonadotropic hypogonadism) have lower perceived social support than control women [9]. | The diagnosis criteria were unclear | ☆ | ☆ | ☆ | ☆  ☆ | interview not blinded to case/control status | ☆ | ☆ | 7 |
| Gibson-Helm et al 2014 Symptoms, health behaviour and understanding of menopause therapy in women with premature menopause [10]. | The diagnosis criteria were unclear | ☆ | ☆ | no description of source | ☆  ☆ | interview not blinded to case/control status | ☆ | ☆ | 6 |
| Schmidt et al 2011 Depression in Women with Spontaneous 46, XX Primary Ovarian Insufficiency [11]. | The diagnosis criteria were unclear | ☆ | no description | no description of source | ☆ | interview not blinded to case/control status | ☆ | non-respondents described | 3 |

**References**

1. Pang Zhenmiao, L. J., & Deng Gaopei (2007). Investigations of personality characteristics and mental health status in patients with premature ovarian failure. *Journal of Clinical Psychosomatic Diseases, 13*(5), 428–430.

2. Kalantaridou, S. N., Vanderhoof, V. H., Calis, K. A., Corrigan, E. C., Troendle, J. F., & Nelson, L. M. (2008). Sexual function in young women with spontaneous 46,XX primary ovarian insufficiency. *Fertil Steril, 90*(5), 1805–1811. doi:10.1016/j.fertnstert.2007.08.040.

3. Benetti-Pinto, C. L., de Almeida, D. M., & Makuch, M. Y. (2011). Quality of life in women with premature ovarian failure. *Gynecological Endocrinology, 27*(9), 645–649.

4. Ji, X. (2013). *Clinical study on the relationship between syndrome types differentiation of TCM and quality of life in premature ovarian failure*. Chengdu: Chengdu University of TCM, CNKI.

5. Yang Li, Z. F., & Dong, Y. (2017). Study on quality of fertility in patients with premature ovarian failure. *Chinese Nursing Research, 31*(1), 115–117.

6. Yela, D. A., Soares, P. M., & Benetti-Pinto, C. L. (2018). Influence of sexual function on the social relations and quality of life of women with premature ovarian insufficiency. *Rev Bras Ginecol Obstet, 40*(2), 66–71. doi:10.1055/s-0037-1615289.

7. Pang, Z. (2006). *The demostration study of the relationship between the social/psychology factors in patients with POF*. Guangzhou: Guang Zhou University of Traditional Chinese Medicine.

8. Davis, M., Ventura, J. L., Wieners, M., Covington, S. N., Vanderhoof, V. H., Ryan, M. E., et al. (2010). The psychosocial transition associated with spontaneous 46,XX primary ovarian insufficiency: illness uncertainty, stigma, goal flexibility, and purpose in life as factors in emotional health. *Fertil Steril, 93*(7), 2321–2329. doi:10.1016/j.fertnstert.2008.12.122.

9. Orshan, S. A., Ventura, J. L., Covington, S. N., Vanderhoof, V. H., Troendle, J. F., & Nelson, L. M. (2009). Women with spontaneous 46,XX primary ovarian insufficiency (hypergonadotropic hypogonadism) have lower perceived social support than control women. *Fertility & Sterility, 92*(2), 688–693.

10. Gibson-Helm, M., Teede, H., & Vincent, A. (2014). Symptoms, health behavior and understanding of menopause therapy in women with premature menopause. *Climacteric the Journal of the International Menopause Society, 17*(6), 8.

11. Schmidt, P. J., Luff, J. A., Haq, N. A., Vanderhoof, V. H., Koziol, D. E., Calis, K. A., et al. (2011). Depression in women with spontaneous 46, XX primary ovarian insufficiency. *Journal of Clinical Endocrinology & Metabolism, 96*(2), 278–287.
