# Supplementary material for: Health-related quality-of-life among patients with premature ovarian insufficiency: a systematic review and meta-analysis
Source: Qual Life Res. 2019 Oct 16;29(1):19–36. doi: 10.1007/s11136-019-02326-2 (PMC6962283; doi:10.1007/s11136-019-02326-2)
Supplement: Supplementary file 4 — Supplementary material 4 (DOCX 23 kb) [file 11136_2019_2326_MOESM4_ESM.docx]

# ESM_4 Abstract translations of Chinese articles

Ref 41. Title: Investigations of personality characteristics and mental health status in patients with premature ovarian failure

Author(s): Pang Zhenmiao, Liang Jing, Deng Gaopi

Journal: JOURNAL OF CLINICAL PSYCHOSOMATIC DISEASES Year: 2007 Issue: 5 Pages: 428-430

Keyword: Premature ovarian failure; Mental health; Personality; Anxiety; Depression;

Abstract:

Objective: To analyze the personality characteristics and mental health status of patients with premature ovarian failure, and to provide a basis for further study of the pathological psychological mechanism of premature ovarian failure.

Methods: A, C type behavior scale, state trait anxiety scale for 79 patients with premature ovarian failure, 80 Patients with polycystic ovary syndrome and 81 healthy controls were tested and analyzed. The blood β-endorphin was tested in patients with premature ovarian failure and healthy controls.

Results: The results showed that the proportion of type A behavior in premature ovarian failure group was significantly higher than that in the group. Cystic ovarian syndrome group and control group; stress and anxiety in depression and depression were strong, and trait anxiety score was higher; time rush, competition, anxiety, depression, and anger factor were significantly higher than the control group (P<0.05 or 0.01). Control, optimism, and social support factors were significantly lower than the control group (P<0.05 or 0.01); time rush, competition, depression, and anger factor were significantly higher than those in the polycystic ovary syndrome group (P<0.05 or 0.01). The level of β-EP was significantly higher than that of the normal group (P<0.01). The scores of anxiety, depression and rational factors in the polycystic ovary syndrome group were significantly higher than those in the control group (P<0.05 or 0.01). Lower than the control group (P <0.05).

Conclusion: Premature ovarian failure patients belong to type A personality trait anxious crowd with mental health problems as anxiety, depression, anger, and many can not be controlled, and should actively take early and effective treatment measures.

Ref 45. Title: Study on quality of fertility in patients with premature ovarian failure

Author(s): Yang Li, Zhou Feijing, Dong Yuezhi

Journal: Chinese Nursing Research Year: 2017 Issue: 1 Pages: 115-117

Keyword: Infertility; Premature ovarian failure; Quality of life on infertility; Influencing factors

Abstract:

Objective: To investigate the conditions and influencing factors of fertility quality of life among infertile women with premature ovarian failure.

Methods: The FertiQol scale was used to investigate 170 cases of premature ovarian failure and 113 healthy women in reproductive medical center. Analyzing the effects of different demographic characteristics on quality of life among patients with premature ovarian failure.

Results: The total score of fertility quality of life (QOL) of infertile women with premature ovarian failure was (54.5±11.89). The five dimensions and total scores of emotional conditions, physical and mental relationship, marriage relationship, social relationship and tolerance were lower than those of healthy women (P < 0.05). The differences were statistically significant (P < 0.05). POF women with older age, primary infertility and no child had significant lower fertility quality of life compared with POF patients with younger age, secondary infertility and children (P < 0.05).

Conclusion: The fertility quality of life among patients with POF is very poor. Active and targeted psychological intervention should be taken early to improve the patients quality of life.

Ref 44. Clinical Study on the Relationship between Syndrome Types Differentiation of TCM and quality of life in Premature ovarian failure

Author(s): Ji Xiujia

Journal: Master thesis from Cheng Du University of Traditional Chinese Medicine Year: 2013 Pages: 1-61

Keyword: premature ovarian failure; quality of life; Syndrome Differentiation; SF-36

Abstract:

Objective: To understand the quality of life of patients with premature ovarian status, this paper discusses the TCM syndrome type and the quality of life, the correlation between traditional Chinese medicine for treatment of ovarian premature aging and carry out the evaluation of clinical curative effect, health education to provide new ideas and methods.

Methods: Adjust the difference of the patients are in Cheng Du University of TCM hospital gynecology clinic patients. All personnel all fill in the school of traditional Chinese medical syndrome of POF questionnaires and health survey short list (SF-36), using cross-sectional survey questionnaire method,114 cases of ovarian premature senility patients and 90 cases of healthy controls for investigation and study, data entry SPSS 17.0 establish database, first check type for frequency distribution analysis, choose frequency distribution larger card type do data processing for card type and life quality comparison.

Results: According to the card type frequency distribution analysis, four frequencies larger card type 100 cases, accounting for 87.7%, and the average age was 35.6±3.96 years old. TCM syndrome type distribution: kidney empty stomach syndrome (36/114), liver and kidney deficiency syndrome (28/114), two spleen and kidney deficiency syndrome (20/114)and Yin deficiency fire (16/114); With this topic into standard of 114 cases of premature ovarian patients and healthy controls were compared, and the quality of life declined obviously, and the liver and kidney deficiency syndrome is the worst.; Different between the uses of statistical significance, spleen and kidney yin deficiency and two than the fire, P <0.05. Physical health (PF,RP, BP, GH) each card type differences, liver and kidney deficiency type lowest score; Mental health (VT,SF,RE, MH) between each card type is indifference ; Premature ovarian patient、quality of life and TCM syndrome type of relationship between certain. Different syndrome types of patients, the quality of life is different, the damage rate of the damaged functions have different emphasis on surface. Four card types in MH more differences, kidney empty stomach type, Yin deficiency type fire more difference (P < 0.05); Four card type in VT score have differences, Yin fire group and spleen and kidney, liver and kidney deficiency two empty two groups of VT score compared (P < 0.0 1); Four card type in RE compare scores have differences, Yin fire group and kidney empty stomach group RE compared to score (P < 0.01); Yin fire group and spleen and kidney two virtual group RE compared to score (P < 0.05); Four card type in RP more differences, Yin fire group and kidney deficiency group compared RP score (P < 0.01).

Conclusion: In patients with premature ovarian with virtual certificate or the actual inclusion card is more common; basic symptoms may be liver and kidney deficiency; The quality of life in patients with premature ovarian declined obviously, and the quality of life in patients with kidney deficiency is the worst; Different syndrome types of different length of spleen and kidney deficiency type of two longest, Yin deficiency type fire shortest; Different syndrome types of patients, the quality of life is different, the damage rate of the damaged functions have different emphasis on surface; This research primarily for the card type between the emphasis, dialectical treatment for clinical provide objective basis.

Ref 46. The demonstration study of the relationship between the social/psychology factors in patients with POF

Author(s): Pang Zhenmiao

Journal: doctoral thesis from Guang Zhou University of Traditional Chinese Medicine Year: 2006 Pages: 1-115

Keyword: POF; Demonstration study; The social/Psychology factors

Abstract:

Objective: The research aims to study the relationship between POF and some socio-psychological factors such as emotional state, personality characteristics, negative life events, etc. by doing an empirical analysis of the collected data, in order to provide supporting evidence of socio-psychological factors for the study of the cause of POF. In addition, this research aims to provide help for the treatment of POF. At the same time, epidemiology study is carried out to find out the cause of POF with the purpose of exploring the risk factors of POF locally. Furthermore, a mathematical model of the morbidity of POF is built to provide theoretical support for the forecasting of POF.

Methods: This research uses the following methods: empirical research, chart measurement, Paper-form surrey, case analysis, mathematical model, etc. The research targets are: the POF group, the normal group and the polycystic ovary group.

According to the design of the research thesis, all research targets were required to fill out Type A Behavioral Chart, Type C Behavioral Chart, STAI, LEU, and self-designed epidemiology survey. All the data are counted and entered into the computer. A database is established to analyze data by SPSS11.5

24 POF patients, 25 infertility patients with obvious anxiety and 21 normal women were selected for the survey as experimental targets. Then, 20ml elbow venous blood were taken from the POF group, the infertility group and the normal group separately and mixed up with test tubes containing 500IU zymofren and ENTA40ul. Next, the mixture was centrifugated for 10 minutes with 2500 rotations/minute at 4° C. The clear liquid on top was taken and put into a fridge of -40° C, waiting to be measured by the β-EP radioactive immunology testing method established by the department of neurobiology of the Number 2 Military Doctors’ University.

Results:

1. The Highly-risk Factors of POF:

It was discovered by chi square experience analysis that the highly-risk factors of POF are as follows: no child-bearing history, family history, living environment containing toxic stuff, smoking, taking contraceptive pills, a history of gynaecological surgery, a history of parotitis, immunological function disease, and a history of tuberculosis. With further Logistic return analysis, we can see that multiple child-bearing, contraceptive pills, high-fat diet, smoking, surgery, parotitis and ovulation-impelling pills are highly-risk factors.

1. The Behavioral Characteristics of POF:

Most people in the POF group have Type A behavioral characteristics. Comparing to the other types, P < 0.05.

1. The Personality Characteristics of POF:

In the category of “Time Haste Feeling”, there is an obvious difference between the POF group and the normal group (P < 0.05). The POF group has stronger time haste feeling than the normal group. Also, there is an obvious difference between the POF group and the polycystic ovary group (P < 0.05). The POF group again has stronger time haste feeling than the polycystic ovary group.

In the category of “Victory Fighting”, there is an obvious difference between the POF group and the normal group (P < 0.05) - the POF group has stronger desire to fight for victory than the normal group. Also, there is an obvious difference between the POF group and the polycystic ovary group (P < 0.05) - the POF group has stronger desire to fight for victory than the polycystic ovary group.

In the category of “Anxiety”, there is an obvious difference between the POF group and the normal group (P < 0.05) - the POF group is more anxious than the normal group. Also, there is an obvious difference between the polycystic ovary group and the normal group (P < 0.05) - the polycystic ovary group is more anxious than the normal group.

In the category of “Depression”, there is an obvious difference between the POF group and the normal group (P < 0.05) - the POF group is more depressed than the normal group. Also, there is an obvious difference between the polycystic ovary group and the normal group (P < 0.05) - the polycystic ovary group is more depressed than the normal group. Moreover, there is an obvious difference between the POF group and the polycystic ovary group (P < 0.05) - the POF group is more depressed than the polycystic ovary group.

In the category of “Anger”, there is an obvious difference between the POF group and the normal group (P < 0.05) - the POF group is angrier than the normal group.

In the category of “Anger Introversion”, there is an obvious difference between the POF group and the polycystic ovary group (P < 0.05) - the polycystic ovary group is angrier and more introvert than the POF group.

In the category of “Rationality”, there is an obvious difference between the polycystic ovary group and the normal group (P < 0.05) - the polycystic ovary group is more rational than the normal group.

In the category of “Control”, there is an obvious difference between the POF group and the normal group (P < 0.05) - the normal group is more in control than the POF group.

In the category of “Optimism”, there is an obvious difference between the POF group and the normal group (P < 0.05) - the normal group ie more optimistic than the POF group. Also, there is an obvious difference between the polycystic ovary group and the normal group (P < 0.05) - the normal group is more optimistic than the polycystic ovary group.

In the categories of “Anger Extroversion” and “Social Support”, there are no obvious differences between the three groups.

1. Does POF display anxiety state?

The chi square test indicates that the POF group belongs to the Anxiety type and was in the anxiety state during the experiment.

Although the polycystic ovary group was also in the anxiety state during the experiment, it doesn’t belong to the anxiety type.

1. POF and Life Events Stimulation

In the category of “Positive Life Events”, there is an obvious difference between the POF group and the other two groups, the normal group and the polycystic ovary group (P < 0.01)- the POF group has less positive life events stimulation than the other two groups.

In the category of “Negative Life Events”, there is an obvious difference between the POF group and the other two groups, the normal group and the polycystic ovary group (P < 0.01)- the POF group has more negative life events stimulation than the other two groups.

In the category of “Total Life Events”, there is an obvious difference between the POF group and the polycystic ovary group (P < 0.01) - the POF group has more total life events stimulation than the polycystic ovary group.

We can see that the POF patients really encounter more negative life events stimulation than the normal group and the polycystic ovary group. Thus, it’s predicted that this finding could be an important triggering factor leading to POF.

1. POF Group’s β-EP Level:

Comparing the POF group with the normal group, β-EP value has an obvious difference (P < 0.01) - the POF group has a much higher β-EP value than the normal group. Comparing the infertility group with the normal group, β-EP value has an obvious difference (P < 0.01) - the infertility group has a much higher β-EP value than the normal group.

1. The Mathematical Model of POF’s Morbidity

The Logistic return model has a predicting function. We can Predict the possibilities of POF by using our acquired mathematical model. Our acquired mathematical model is: In(*P*/1-*P*) -0.573 + 1.220*X*_1_ -1.725*X*_2_ +1.983*X*_3_-0.991*X*_4_ +1.835*X*_5_+2.398*X*_6_ -0.026*X*_7_+2.666*X*_8_ + 3.764*X*_9_+1.361*X*_10_ + 4.215*X*_11_ + 3.745*X*_12_ - 2.345*X*_13_ +3.749*X*_14_

We can obtain the morbidity of POF by bringing the female- related conditions into the formula. If the probability is very small, we can infer that she won’t get POF. If the probability is greater than 0.5, we can suspect that she will get POF. Then we’d suggest her to take some tests and reduce the frequency of some highly-risk factors in her life, for example, reducing multiple child-bearing, smoking, taking contraceptive pills, taking ovulation-impelling pills, reducing the chances of induced abortion and gynaecological surgery. A balanced and light diet is advocated to diminish the morbidity of POF.

Conclusions: Through empirical research, it’s found that POF patients mostly have Type A behavioral characteristics and anxiety type. They were in the anxiety state during the experiment and went through more negative stimulation than other people in their lives. These negative emotions continuously interfere patients’ energy - nerve - endocrine system, i.e. hypothalamus - pitutary - ovarian axle. Then, they have a further influence of the hypothalamus’ FSH, LH and ovary’s E2 secretion. Therefore, it’s demonstrated that POF is a disease of both body and soul and it reminds us that we need to pay attention to the curing of the patient’s emotional problems in the clinical practice in order to achieve twice the result with half the effort.
