# Supplementary material for: Health-related quality-of-life among patients with premature ovarian insufficiency: a systematic review and meta-analysis
Source: Qual Life Res. 2019 Oct 16;29(1):19–36. doi: 10.1007/s11136-019-02326-2 (PMC6962283; doi:10.1007/s11136-019-02326-2)
Supplement: Supplementary file 5 — Supplementary material 5 (DOCX 25 kb) [file 11136_2019_2326_MOESM5_ESM.docx]

ESM_5 Summary of the questionnaires used in the studies included in the meta-analysis

| Test | World Health Organization QoL (WHOQoL-BREF) | 36-Item Short Form Survey from the RAND Medical Outcomes Study | Type A/C behavior pattern (TABP)(TCBP) | Female Sexual Function Index (FSFI) | Derogatis Interview for Sexual Function (DISF-SR—Female Version) | International Fertility Quality of Life Questionnaire (FertiQoL) |
| --- | --- | --- | --- | --- | --- | --- |
| Author | WHO-QoL group | EUROQoL Group | Chinese National Collaborative Study Group. (based on Jenkings Activity Scale) | Rosen R. R. Wood Johnson Medical School, USA | Derogatis 1978 | Cardiff Fertility Studies Research Group |
| Chinese Language Adaptation | Hong-Kong & Taiwanese versions 28 items. Quality check | Simplified Chinese. Quality check. | Simplified Chinese. Commonly used in China since 1980s Adapted by Zhang | Simplified Chinese version by the first author. Quality check. | Simplified Chinese by the second author. Quality check | Simplified Chinese by the translation team. Translation verified by local bilingual fertility experts. |
| Study used /country | Yela, et al 2018 [1]. / Brazil Benetti-Pinto et al 2011 [2]. / Brazil | Ji 2013 [3]. / China | Pang et al 2007 [4]. / China | Yela, et al 2018 [1]. / China | Kalantaridou et al 2008 [5]. / USA | Yang et al 2017 [6]. / China |
| Purpose of development | Generic HrQoL / self-report | Generic HrQoL / self-report | Assessment of behaviour patterns | Satisfaction with sexual activity | Quality of sexual functioning | Fertility quality of life in people experiencing fertility problems. |
| Disease stage | All stages | All stages | All stages | All stages | All stages | All stages |
| Age range | Adult female and men | Adult female and men | Adult female and men | Adult Female | Adult Female | Adult female and men |
| Response options / score range | 5 point Likert scale transformed to 0-100 (worst-best) | Various Likert scales summated scores transformed to 0-100 scale (worst-best) | TABP dichotomous questions summed (low=Type B; high = Type A)  TCBP 4 point Likert | 6 point Likert scale | Combination of 9- and 5-point scales. High scores = adequate knowledge, sexual function, and liberal attitudes. | 5 point Likert 0–4. Transformed to 0 to 100 scales (worst-best). |
| Time framework | Last 2 weeks / a longer timeframe is possible | Last 4 weeks in the standard version. | No timeframe | Last 4 weeks | No timeframe | No timeframe |
| Number of domains | 4 | 8 | 3 | 6 | 10 | 4 |
| Domains measured | Social, Emotional, Physical, Environmental + 2 questions concerning perception of HrQoL and general health | Physical, Role limitations, Bodily pain, Social, General mental health, Role limitations /emotional, Vitality (energy/fatigue), Gen health. + 1 item on health transition | TU (time urgency), CH (competitive hostility), L (lie) | Desire, Arousal, Lubrication, Orgasm, Satisfaction, Pain | Information, Experience, Drive, Attitudes (Liberal), Psychological, Affects (Pos & Neg), Gender role, Fantasy, Body Images, Satisfaction | Core and Treatment-related QoL + overall life and physical health. Domains: Emotional, Mind–body, Relational; Social. |
| Respondent burden | English 26 items HK=28 / Taiwan=28 * | 36 items | 60 items | 19 items | 26 items | 36 items / 24 in core set |
| Reliability | Cronbach’s α .59 to .78. Test-retest ICC all above 0.78 | Cronbach’s α 0.85 to 0.87 | Cronbach’s α 0.64, and 0.65 in matched case–control study. | Cronbach’s α 0.84 Good test–retest reliability. | Cronbach's α.56 to .97 Test-retest between >.77 to >.90 | Cronbach's α 0.72 to 0.92. |
| Validity | Discriminate validity good | Factor loadings were similar to those found in the U.S. population. Construct validity confirmed by known groups. | Discriminate validity of injury /non-injury youth. | Concordance with Locke-Wallace Marital adjustment test. Good discriminate validity | Discriminates between sexual disorder and normal | Sensitivity analyses showed expected relations between QoL and gender, parity and support-seeking. |
| Alternative Forms | paper, telephone, face-to-face, spoken Cantonese | paper | paper | paper | paper | paper |
| Instrument search algorithm | (WHOQoL-BREF[title]) AND (Chinese OR Cantonese OR China OR Hong Kong OR Shanghai) | (SF-36[title] OR SF36[title]) AND (Chinese OR Cantonese OR China OR Hong Kong OR Shanghai) | (TABP) AND (Chinese OR Cantonese OR China OR Hong Kong OR shanghai) | (FSFI) AND (Chinese OR Cantonese OR China OR Hong Kong OR shanghai) | (Derogatis) AND (Chinese OR Cantonese OR China OR Hong Kong OR shanghai) | (FertiQoL) AND (Chinese OR Cantonese OR China OR Hong Kong OR shanghai) |
| manuscripts found | 164 | 71 | 5 | 41 | 4 | 5 |
| Main references for Chinese translation | HK version: Leung, et al. Qual Life Res (2005) 14: 1413. [6]  Taiwan version: Yao et al J Formos Med Assoc. (2002) 101(5):342-51 [7] | Lam, et al (HK) version. J Clin Epidemiol, (1998), 51,1139–1147 [8] | Zhang. Acta Psychologica Sinica. 1985, 314–321 [9] | Sun et al. J Sex Med. (2011) 8(4):1101-11. Epub [10] | Tang, et al. Archives of sexual behavior, (1997), Vol.26(1), pp.79-90 [11] | Seen-tsing & Wai-ming Human Fertility, (2016) 19:4, 268-274 [12] |

* Chinese items “Do you feel respected by others?”, and “Are you usually able to get the things you like to eat?” * note different domain allocations for these additions in HK & Taiwan version

**References**

1. Yela, D. A., Soares, P. M., & Benetti-Pinto, C. L. (2018). Influence of sexual function on the social relations and quality of life of women with premature ovarian insufficiency. *Rev Bras Ginecol Obstet, 40*(2), 66–71. doi:10.1055/s-0037-1615289.

2. Benetti-Pinto, C. L., de Almeida, D. M., & Makuch, M. Y. (2011). Quality of life in women with premature ovarian failure. *Gynecological Endocrinology, 27*(9), 645–649.

3. Ji, X. (2013). *Clinical study on the relationship between syndrome types differentiation of TCM and quality of life in premature ovarian failure*. Chengdu: Chengdu University of TCM, CNKI.

4. Pang Zhenmiao, L. J., & Deng Gaopei (2007). Investigations of personality characteristics and mental health status in patients with premature ovarian failure. *Journal of Clinical Psychosomatic Diseases, 13*(5), 428–430.

5. Kalantaridou, S. N., Vanderhoof, V. H., Calis, K. A., Corrigan, E. C., Troendle, J. F., & Nelson, L. M. (2008). Sexual function in young women with spontaneous 46,XX primary ovarian insufficiency. *Fertil Steril, 90*(5), 1805–1811. doi:10.1016/j.fertnstert.2007.08.040.

6. Leung, K. F., Wong, W. W., Tay, M. S. M., Chu, M. M. L., & Ng, S. S. W. (2005). Development and validation of the interview version of the Hong Kong Chinese WHOQOL-BREF. *Quality of Life Research An International Journal of Quality of Life Aspects of Treatment Care & Rehabilitation, 14*(5), 1413–1419.

7. Yao G , C. C. W., Yu C F , et al. (2002). Development and verification of validity and reliability of the WHOQOL-BREF Taiwan version. *Journal of the Formosan Medical Association 101*(5), 342.

8. Lam CL, G. B., Ren XS, et al. ( 1998). Tests of scaling assumptions and construct validity of the Chinese (HK) version of the SF-36 health survey. . *J Clin Epidemiol, 51*, 1139–1147.

9. Zhang, B. (1985). PSYCHOPHYSIOLOGICAL REACTION IN CARDIOVASCULAR DISEASE: II. A STUDY ON THE BEHAVIOR PATTERN OF CORONARY HEART DISEASE PATIENTS. *Acta Psychologica Sinica, 17(3)*, 314–321.

10. Sun X., Li C., Jin L., Fan Y., & Wang D. (2011). Development and validation of Chinese version of female sexual function index in a Chinese population—A pilot study. *J Sex Med, 8*(4), 1101–1111. doi:10.1111/j.1743-6109.2010.02171.x.

111. Tang, C. S., Lai, F. D., & Chung, T. K. (1997). Assessment of sexual functioning for Chinese college students. *Arch Sex Behav, 26*(1), 79–90.

12. Lo, S. S., & Kok, W. M. (2016). Sexual functioning and quality of life of Hong Kong Chinese women with infertility problem. *Hum Fertil (Camb), 19*(4), 268–274. doi:10.1080/14647273.2016.1238516.
